# Prognostic impact of tumor budding and EMT in periampullary adenocarcinoma: a quantitative approach

**DOI:** 10.7150/jca.46093

**Published:** 2020-09-17

**Authors:** Éva Kocsmár, Gábor Lotz, András Kiss, Markus Hoerner, Ekaterina Petrova, Nikolaus Freudenberg, Ágnes Csanádi, Birte Kulemann, Martin Werner, Peter Bronsert, Ulrich Friedrich Wellner

**Affiliations:** 1 2nd Department of Pathology, Semmelweis University, Budapest, Hungary.; 2 Institute of Surgical Pathology, University Medical Center, Freiburg, Germany.; 3 Tumorbank Comprehensive Cancer Center Freiburg, Medical Center - University of Freiburg, Germany.; 4 Faculty of Medicine, University of Freiburg, Germany.; 5 German Consortium for Translational Cancer Research, Freiburg, Germany.; 6 Department of Surgery, UKSH Campus Lübeck, Germany.; 7 Department of Surgery, University Medical Center, Freiburg, Germany.

## Abstract

The presence of invasive cell clusters known as tumor budding and the closely related epithelial mesenchymal transition (EMT) have a prognostic impact on cancer patients' overall survival. Interestingly, data quantitatively analyzing and correlating the amount of tumor buds and patient overall survival as well as the impact of expression of epithelial phenotype markers are missing.

Periampullary carcinoma samples of 171 patients were immunohistochemically stained for E-Cadherin (ECad). Tumor cell clusters (TCC, defined from one to 50 cells) were manually quantified comprising tumor cell number and subcellular localization of ECad expression (membranous, cytoplasmic or mixed). Data analyses were performed using elastic net feature selection. Hereby, five distinct intervals of TCC sizes and corresponding fractions of cells with distinct ECad expression were identified. Prognostic features of the defined budding categories were entered into a subsequent Cox regression model together with standard clinicopathological parameters and, based on the model prediction, cases were categorized into “low and high budding” grades.

Overall median TCC size was 16 cells (range: 2-36 cells). The median number of TCCs per tumor was 42 (range: 3-283). Elastic net feature selection identified TCCs of 6-10 and 31-35 cells as prognostically most relevant negative and positive features, respectively. Regarding ECad expression, cytoplasmic ECad expression in TCCs of 11-15 as well as of 26-30 cells revealed prognostic relevance. Combining TCC numbers and ECad expression, budding grade qualified as independent prognostic factor for patient overall survival (*p<*0.001) in a multivariable clinicopathologic Cox model.

Applying an advanced modelling by machine learning on a cohort of periampullary cancers, we show that not the smallest TCCs (1-5 cells) but tumor cell nests containing 6-10 cells display the strongest negative prognostic relevance. Moreover, we demonstrate that larger TCCs might have a strong positive prognostic impact in periampullary adenocarcinomas, contributing to establishing an advanced grading system.

## Introduction

The term “tumor budding” was first introduced by Imai et al. 60 years ago 1. During the next decades tumor budding - defined as a cohesive complex of up to five tumor cells detached from the main tumor mass 2,3 - has been identified as an important prognostic histological cancer characteristic 4. Strong tumor budding correlates directly with tumor aggressiveness (poor differentiation and tumor stage) and subsequently with patients overall survival 5,6. Furthermore, tumor budding represents a histomorphological manifestation of epithelial to mesenchymal transition (EMT) 7-9.

Several studies investigated the prognostic value of tumor budding, focused on tumor cell clusters containing five or less tumor cells 3,4,9. Interestingly, histological studies quantifying and characterizing tumor buds in their complex dimensionality (comprising EMT characteristics and cell counts) in correlation with clinicopathological data are missing. The amount of tumor cells defining a tumor bud is currently also not standardized 3,9-12. The first harmonization by the International Tumor Budding Consensus Conference (ITBCC) group defines the cut-off value for a tumor bud as up to four cells 13. Besides the term tumor bud, a novel histopathological cell cluster entity “poorly differentiated cluster (PDC)” was established. Herein, PDCs - defined as five or more cells - also demonstrate a negative impact on patients overall survival 14,15. Nevertheless, both tumor bud and PDC do not result from a quantitative systematic analysis but rather from a historical basis.

The term periampullary carcinoma comprises a group of tumors with a common embryological origin, originating from the same part of the foregut 16. Due to the topographical proximity and the anatomical complexity of the periampullary region, the primary tumor origin (ampulla Vateri, duodenum, pancreatic head and distal bile duct) often cannot be exactly determined during macroscopic and microscopic examination. In this case, surrogates for the topographical tumor allocation and the consequent prognostic and predictive TNM classification might be specific for the precursor lesions in the affected organ 17-20.

The aim of this study was a quantitative morphomic analysis of tumor cell clusters and their ECad staining pattern in periampullary adenocarcinomas using a well characterized cohort 20.

Therefore, we applied a quantitative statistically based approach by linking tumor budding, EMT features and patient prognosis in periampullary adenocarcinomas. Hence, our study characterizes these neoplasms on the basis of several features of the complex morphological appearance of tumor development (morphome) 21,22 in a comprehensive way. Furthermore, the applied morphome model delineates a potential strategy of risk stratification in periampullary carcinomas, of which the origin cannot be reliably ascertained.

## Materials and Methods

### Patients and clinical data

The study protocol was in accordance with the Declaration of Helsinki and was approved by the Ethics Committee of the University Medical Center Freiburg (Ref: 13/11). Patient selection, exclusion criteria, handling and processing of clinical data, surgical processes and pathological workup are described in detail by Bronsert et al. 20. Briefly, patients who were included in the study underwent Whipple procedure or - in case of positive intrapancreatic resection margin - total pancreatectomy at the Clinic for General and Visceral Surgery, University of Freiburg between 2001 and 2011 due to one of the following periampullary adenocarcinomas (PAMPAC): pancreatic head ductal (PDAC), extrahepatic distal bile duct (DBDAC), ampullary (AMPAC) and duodenal (DUOAC) adenocarcinoma. Patients with perioperative mortality, follow-up time below one month and insufficient tissue material for ECad staining were excluded from the study (27 cases). Histological workup was performed at the Institute for Surgical Pathology, University of Freiburg.

### Staining methods and evaluation

All PAMPAC were re-reviewed for tumor budding using standard hematoxylin and eosin (H&E) stained tissue slides. Herein, the slides containing the tumor areas with the highest number of tumor buds were identified. Corresponding tissue blocks were selected and processed further for ECad immunohistochemical staining. Two µm-thick tissue sections were prepared and stained for ECad: Heat-induced antigen retrieval was performed in pH 9.0 antigen retrieval buffer (S2368; Dako, Hamburg, Germany) at 95°C for 40 min. ECad primary antibody (E-Cad, IR059; Dako), respectively with LINKER reagent (K8021; Dako) was used on an Autostainer LINK 48 (Dako) device. For the Streptavidin-biotin peroxidase detection, EnVision® Flex Peroxidase-Blocking Reagent (SM801, Dako), EnVision Flex+ Mouse (LINKER, K8021; Dako) secondary antibody and EnVision Flex/HRP solution (SM802, Dako) were used. Omission of the primary antibody served as negative control. Normal epithelium was used as internal positive controls for ECad. Hematoxylin was used as counterstaining before adding the coverslip.

**Figure 1:**
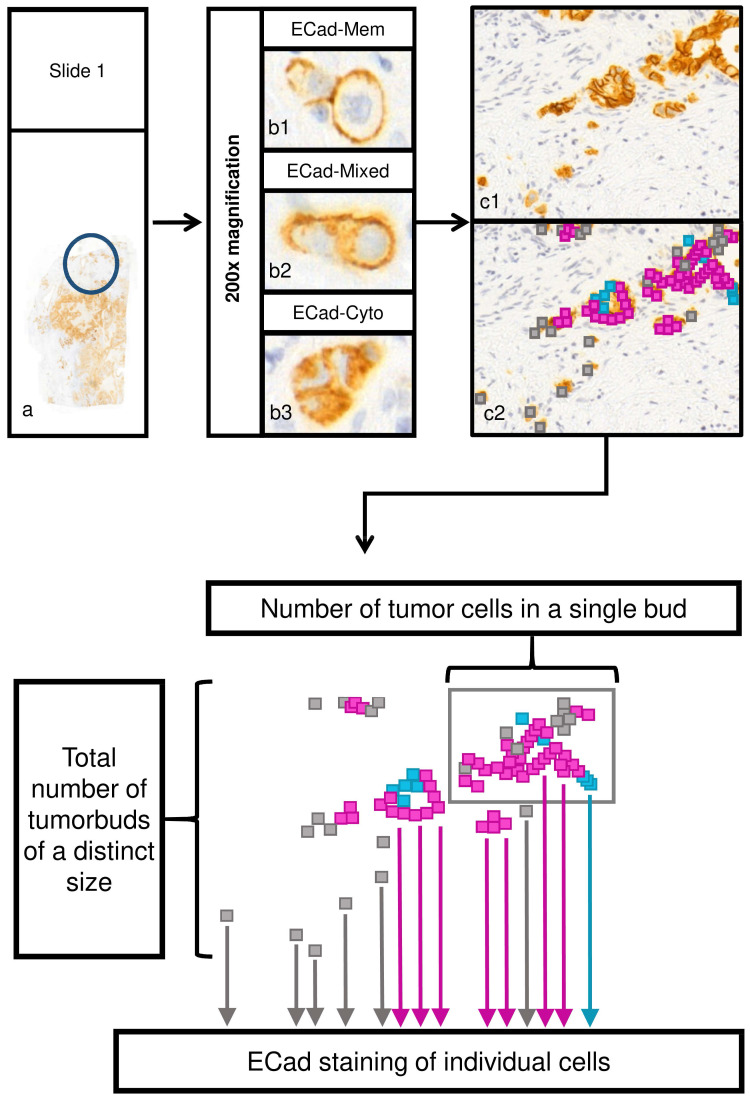
Evaluation process of tumor cell cluster features and subcellular localization of ECad. a) Assessment of tumor budding in two representative areas in each tissue sample, respectively (original magnification: 200x). To assess tumor budding and EMT status in the bud-forming cells simultaneously, ECad expression was determined by immunohistochemistry at the invasion front at the single cell level. Every tumor cell cluster of size ≤ 50 tumor cells in the given area was investigated and ECad expression pattern was recorded for each tumor cell. b) Subcellular localization of ECad was recorded as the following: membranous only (b1); mixed (both cytoplasmic and membranous (b2); cytoplasmic only (b3). Original magnification: 200x. c) Example for a representative area (c1). Different subcellular localization types of ECad are represented by color codes in the cells (c2); membranous: blue, cytoplasmic: grey, mixed: pink. (Original magnification: 200x) d) Schematic representation of evaluated morphome features. ECad staining of individual cells, number of tumor cells in each single tumor cell cluster and total number of tumor cell clusters of a distinct size were recorded. ECad=E-cadherin.

Next, each slide was digitalized using the Mirax Scan Panoramic scanner (3DHistech, Budapest, Hungary) with a 20× objective. Previously identified tumor areas with high budding at the invasion front were verified, and one representative field of 200-fold magnification (field area: 516788.1µm^2^) was evaluated using CaseViewer 2.0 software (3D Histech, Budapest, Hungary). In the studied region, isolated tumor cells and cell clusters comprising ≤50 cohesive tumor cells were the subject of evaluation. Each of these tumor cells was separately investigated and its ECad expression pattern (membranous, cytoplasmatic, mixed; **Figure 1**) was determined. Tumor cell number of each cohesive cluster was quantified in a range from 1 cell to 50 cohesive cells.

### Morphomic features

In all observed areas, cohesive tumor cells (defined as tumor cell clusters) were quantified according to their tumor cell amount and ECad expression. The maximal tumor cell number for one cluster to be defined as cluster and not as main tumor mass was 50 tumor cells, the smallest cluster one cell. Tumor clusters were assigned to ten different cell cluster size categories: 1-5, 6-10 … and 46-50 cells, respectively. Subcellular ECad expression was integrated into the dataset as relative fraction of tumor cells to all cells of the clusters showing membranous, mixed or cytoplasmic ECad staining (**Figure 1**).

### Statistical methods

To identify the prognostic morphome features among the numerous variables, feature selection was performed using elastic net regression 23 to reduce the number of noisy features and to improve the parameter-sample size ratio. The morphomic feature matrix consisted of the absolute cell cluster count in the size categories and the relative fractions of cells expressing ECad in a cytoplasmic, mixed and membranous pattern. Elastic net regression was performed using the glmnet package 23 with the Cox proportional hazards model. 24 To account for eventual instability, feature selection was repeated ten times with tenfold internal cross-validation and an alpha value of one. Features that were consistently and repeatedly (>80%) selected by the elastic net (non-zero coefficient under the optimal lambda tuning value) were used for further analysis.

For interpretation and evaluation of the selected morphomic features, all selected features were entered into a Cox regression model for predicting patients overall survival. The linear predictors from this model were categorized into two equally sized groups named “low and high budding grade”. The prognostic value of these categories was assessed by Kaplan-Meier plot and log rank test. In a last step, a final Cox proportional hazards model with stepwise selection was used to identify histopathological variables that independently influenced the overall survival (OS) in the whole patient cohort as well as in the PDAC subgroup.

Differences between the low and high budding grade categories regarding histopathological parameters were compared by Fisher's exact test. Tumor cell cluster size correlations were analyzed using inverse regression. All calculations and plotting were performed using R software version 3.4.4. (www.r-project.org;R Core Team 2017). Statistical testing was performed two-sided at a significance level of *p<*0.05.

## Results

### Patient cohort

171 patients operated between 2001 and 2011 at the University Medical Center Freiburg were included (**Table 1**). Gender distribution was 88 males and 83 females. Median age was 67 years (range 30-89 years). The tumors were classified according to their origin (110 pancreas ductal adenocarcinomas (PDAC)/36 ampullary carcinomas (AMPAC)/seven small intestinal carcinomas (including duodenal adenocarcinomas, DUOAC)/18 distal bile duct carcinomas (DBDAC)). Tumors were subdivided into morphological subgroups according to their histological subtype (34 intestinal/104 pancreatobiliary/11 mixed (intestinal-pancreatobiliary)/17 (undifferentiated) according to Albores-Saavedra et al. 25 Tumors with rare histomorphology were categorized as “other” (5 cases). Non adenocarcinomas (e.g. neuroendocrine, mesenchymal tumors etc.) were excluded (detailed cohort characteristics were previously described) 20.

According to the 7^th^ TNM classification 26 7.6% (13 cases) of the patients were classified as T1, 15.8% (27 cases) as T2, 66.1% (113) as T3 and 10.5% (18 cases) were at T4 stages. Tumor positive lymph nodes (N+) were detected in 114 cases (66.7%). Three tumors (1.8%) were graded as well differentiated (G1), 108 (63.2%) tumors as moderate differentiated (G2), 59 (34.5%) tumors as undifferentiated (G3) and one (0.6%) tumor as dedifferentiated (G4). Lymphovascular invasion was present in 74 cases (43.3%) while blood vessel invasion was observed only in 26 cases (15.2%). Median follow-up was 16 months (ranged 1-116 months).

### Tumor cell cluster sizes and subcellular staining pattern of E-cadherin expression

In total, n=144395 cells were assessed manually and evaluated according to their E-cadherin (ECad) expression. Considering ECad staining, 36981 (26%) tumor cells demonstrated a membranous, 77535 (54%) tumor cells a mixed and 29879 (21%) tumor cells a cytoplasmic pattern. The median number of tumor cell clusters per field of 200-fold magnification was 42 (range 3-283). The median total cell number of cells in the clusters was 336 (range 16-1108) (more details given in **Table 1**). Median cell cluster size was calculated individually for each patient and ranged from 2 to 36 cells, with an overall median of 16 cells across all tumors. There was no statistically significant relationship between total cell count, cluster count or cluster size and clinicopathological parameters (tumor location, T/N/M stage, tumor grade, lymphangiosis carcinomatosa, blood vessel infiltration, and margin status) in linear regression analysis (*p*>0.05; data not shown).

**Figure 2:**
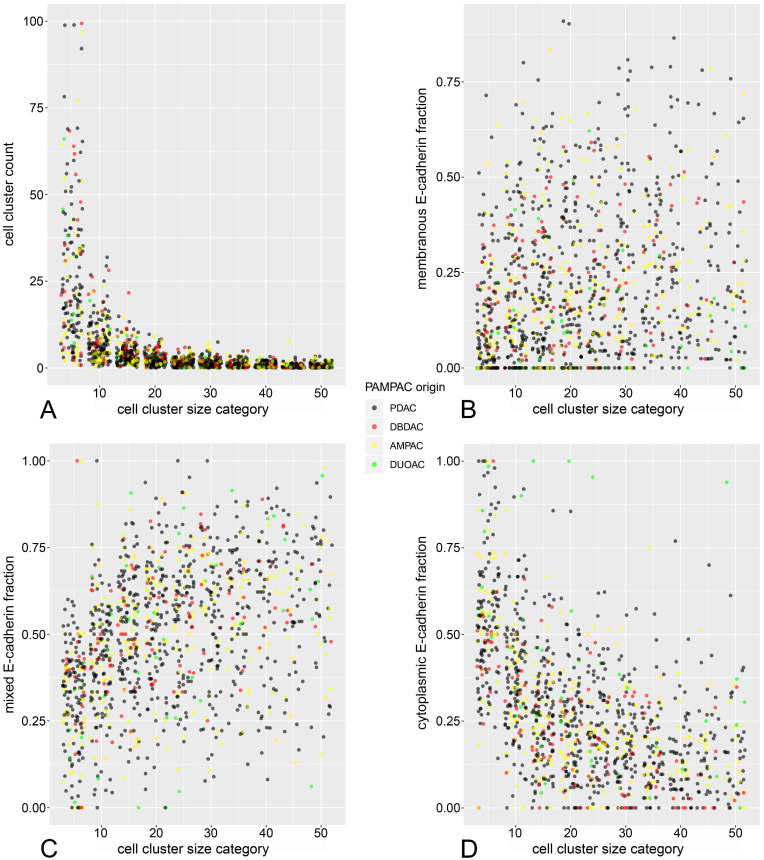
Jitter plots of cell cluster size versus cluster count (A), fraction of cells with cytoplasmic (B), mixed (C), and membranous (D) E-Cadherin staining pattern.

As described above, cell cluster counts were summarized in ten cluster size categories (1-5, 6-10 … and 46-50 cells). Relative frequencies of cells in each cluster category for membranous, mixed and cytoplasmic ECad staining were calculated (Supplementary Table 3A-E). Hereby plotting cluster count versus cluster size (**Figure 2A**) revealed a significant inverse relationship (count ~ 1/size, *p<*0.001 in inverse regression), meaning that cell cluster number increases steeply with decreasing cell cluster size.

Regarding the subcellular ECad expression pattern, similar inverse proportional relationships to cell cluster size could be demonstrated (**Figure 2B-D**). Herein, the fraction of cells with cytoplasmic staining increased significantly in smaller cell clusters while the fraction of cells with mixed and membranous pattern decreased with decreasing cluster size. These observations are reminiscent of a previous analysis from our working group 27, with the notable difference that in the present study, these relationships can be demonstrated in terms of a between-subjects analysis across many tumors.

### Morphome feature selection

Elastic net feature selection identified the counts of following cell cluster size categories as predictors of overall survival: 6-10 cells, 31-35 cells, and the fractions of cytoplasmic ECad expressing cells in clusters of sizes 11-15 as well as 26-30 cells. A Cox model containing only the selected features (**Table 2**) shows that clusters of 6-10 cells and cytoplasmic ECad expression in clusters of 11-15 as well as 26-30 cells are negative prognosticators (hazard ratio >1) for overall survival. Notably, higher counts of tumor cell clusters with 31-35 cells had a positive influence on patients overall survival (hazard ratio <1). All these morphome features were statistically independent prognostic factors (*p<*0.05). To simplify further analysis, linear predictors derived from this multivariable model were used to stratify patients into two equally sized quantile groups termed “low budding grade” and “high budding grade”, with low and high predicted risk of death during follow-up, respectively. Univariable comparisons of low versus high budding grades are shown in Supplementary Table 2.

### Prognostic value of tumor budding grade in multivariable analysis

As the final step, multivariable survival modelling (**Table 3**) including baseline clinicopathological parameters (age, sex, T-stage, lymph node status, grade, lymphovascular, blood vessel and perineural invasion, histologic subtype and tumor origin) together with the budding grade (defined above) was performed. Cox proportional hazards model including all PAMPAC patients identified distant metastasis (HR 3.856, 95% CI 1.519-9.792, *p=*0.005), intestinal histologic subtype (HR 0.154, 95% CI 0.052-0.458, *p=*0.001), ampullary location (HR 0.42, 95% CI 0.215-0.822, *p=*0.011) and high budding grade (HR 2.606, 95% CI 1.653-4.108, *p<*0.0001) as independent prognostic factors regarding patients overall survival.

As the majority of the included patients (110 cases, 64%) suffered from PDAC, we performed the same analysis in the subgroup of PDACs. In PDAC, metastatic spread to the regional lymph nodes (HR 2.11, 95% CI 1.1-4.03, *p=*0.025) as well as metastatic spread to distant organs (HR 3.19, 95% CI 1.09-9.36, *p=*0.035) and high budding grade (HR 3.46, 95% CI 1.91-6.26, *p<*0.0001) demonstrated an independent prognostic value on patient overall survival (**Table 4**).

**Figure 3:**
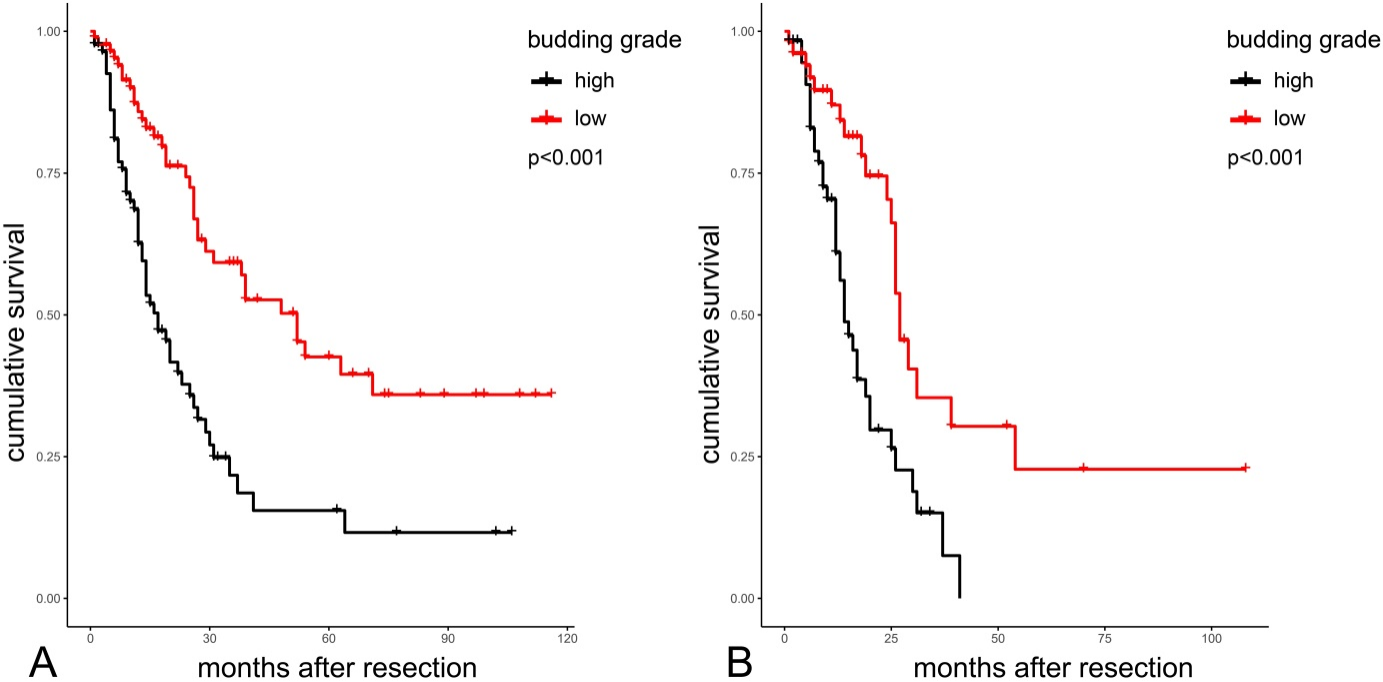
Kaplan-Meier analysis of budding grades in the total cohort (A) and in PDACs (B) PDAC: Pancreatic Ductal Adenocarcinoma.

Survival plots highlighting the significant differences in overall survival between the two budding grade groups in the whole PAMPAC cohort are shown in **Figure 3A.** Median overall survival was 52 vs. 17 months in patients with low versus high budding grade (*p<*0.001, **Table 5**). In the subgroup of PDAC, median survival was 27 vs. 14 months in patients with low versus high budding grade (*p<*0.001 **Table 5**).

## Discussion

In the current literature, tumor buds are commonly defined as an isolated cancer cell or cluster including less than or equal five 2 or less than or equal four 3 cancer cells. The prognostic value of budding in colorectal cancer is recognized by the Union for International Cancer Control (UICC) 31,32 and the European Society for Medical Oncology consensus guidelines suggest budding as a potential prognostic factor in early colorectal carcinoma 31.

Albeit EMT is a pivotal process in cancer biology, the link between the EMT and tumor budding - as a putative histomorphological manifestation of EMT- is still not well clarified. The concept of EMT describes a process in which tumor cells disengage from the main tumor mass by activating transcriptional programs 33 and subsequently changing their protein-expression pattern and behaviour towards a motile mesenchymal phenotype 27,28,29,34. EMT represents a hallmark of cancer metastasis, involving decreased levels of epithelial cell adhesion proteins and increased levels of mesenchymal markers 35. Herein, the shift of membranous to mixed/cytoplasmic ECad expression is considered as a key feature of EMT 36-38.

To investigate the prognostic impact of tumor budding and ECad shuttling simultaneously, we performed quantitative assessment of tumor cell cluster size and immunohistochemical ECad expression pattern of the tumor cells from 171 resected periampullary carcinomas. Based on the detailed tumor budding analysis comprising tumor cell quantification, cell cluster assignment and subcellular ECad expression, using elastic net feature selection and multivariable survival modeling, prognostic histologic features were identified from this morphomic dataset.

The morphome features with the strongest significant impact on patient overall survival were counts of tumor cell cluster of a size from six to ten cells (poor prognosis) and of a size from 31 to 35 cells (good prognosis). Of note, the final statistical model included all compiled standard clinicopathological parameters as well as the histological subtype (as published previously) 20.

Several studies were conducted to identify prognostic subgroups in different carcinomas according to their tumor budding behavior 3,4. These studies aimed to establish classifications which can be implemented in routine diagnostic protocols. As a drawback (from our point of view) all studies were semi-quantitative and disregarded EMT characteristics. By integrating a quantitative high dimensional modelling for the description of complex tumor budding patterns comprising ECad shifting in periampullary carcinomas, our data present a unique approach for analyzing the effects of tumor budding and EMT. In their relationship, EMT leads to cell cycle attenuation in the tumor cells resulting in low potential for division and mass formation, reduced effectiveness of chemotherapeutic agents but maintained invasive properties 5,39-41. Consequently, EMT shifts the balance from formation of larger cell clusters to development of smaller ones (tumor buds) with high migration potential. Hereby, our results also draw attention to the importance to assess the amount of larger cell clusters and to investigate their role as a positive prognosticator in routine histopathology diagnostics.

Albeit the strong relationship between EMT and tumor budding is widely accepted, verification of direct quantitative correlations between these phenomena is still scanty. ECad is a transmembrane adhesion glycoprotein 38,42. During EMT, ECad shuttles from the cell membrane to the cytoplasm. ECad shuttling correlates with a decreased cancer differentiation 27,29,34. Therefore, EMT shifts the balance from the formation of large cell clusters to the formation of small cell clusters (tumor buds) with a higher migration potential 27,29. Our previous study supported that ECad shifting from the membrane to the cytoplasm correlates inversely with the size of the cell cluster assessed two-dimensionally 27. Descriptive histologic data of bud sizes and different ECad staining patterns strongly support our previous findings, as jitter plots (**Figure 2**) show the membranous and mixed pattern to be more characteristic for the larger clusters and the cytoplasmic ECad staining for the smaller buds. The loss of membranous ECad expression indicates EMT which is related to increased tumor cell dissemination, tumor spread and decreased patient overall survival 27,28,30,43. Our morphome features strengthen this since tumor cell clusters expressing ECad in cytoplasmic pattern had negative prognostic impact on the patients overall survival (**Table 2**). These data are in accordance with our prior study, indicating membranous ECad positivity as a positive prognostic factor in pancreatic cancer. 30 Furthermore, we found smaller tumor buds not only to be characterized by shifting of ECad from the membrane to the cytoplasm but also to be a negative prognosticator of patient survival. On the contrary, presence of larger tumor cell clusters was associated with better overall survival. Besides the prognostically relevant aspects, ECad represents a straightforward applicable, reliable and available immunohistochemical marker, which is used (e.g. for differential diagnosis between lobular and invasive ductal carcinoma of the breast) by virtually all histo-pathological institutes. Simultaneously, ECad comprises the ability to a) embody the tumor cell EMT- status 38,44,45 and b) to be a specific marker for epithelial tumor cells. By incorporating this dual role, the application of an ECad staining is superior to using an epithelial only specific marker (like Cytokeratin) or a mesenchymal only specific marker (like Vimentin) respectively. Besides the fact that the overwhelming majority of tumor cells is E-Cadherin positive, characteristic “shifting” of ECad (changes of expression according to subcellular localization) allows us to analyze the whole process of epithelial-mesenchymal transition on the single cell level.

It is important to emphasize that tumor budding is a special, incomplete form of EMT since tumor buds will not acquire complete mesenchymal phenotype (for example they lack to express vimentin) 6,30,46. Accordingly, mixed, moreover membranous expression was also found in considerable fraction of cancer cells of smaller nests, as some cohesive interaction is still needed to maintain integrity of these cell clusters. Therefore, ECad shifting is an important indicator of partial EMT while tumor budding is the histologic equivalent of this process. Our previous study showed that about 95% of the tumor bud forming cells were ECad positive 29. Hence, the detection of ECad shifting represents an adequate surrogate to detect tumor budding and characterize EMT via immunohistochemistry 29,30. The association between partial EMT and ECad shifting was also strongly supported by our survival data, as cytoplasmic expression type of ECad was revealed as negative prognostic factor in our study, even in larger tumor cell clusters (cell cluster size 26-30).

Tumor budding is widely associated with bad prognosis. Interestingly, not only the lack of smaller tumor buds, but also the presence of larger tumor cell clusters have a positive prognostic effect on patients overall survival. To our knowledge, the presented study is the first one describing larger tumor cell clusters as positive prognosticators in periampullary tumors. These results should be analyzed in further studies also comprising other cancer entities.

Furthermore, our data emphasize that the quantitative concept of tumor grading can be extended and refined by integrating EMT relevant data. Hereby, patients with periampullary carcinomas but without the knowledge of the exact topographical primary tumor can be stratified into prognostically relevant groups and, where applicable, benefit from an adjusted adjuvant treatment. Due to the fact that the digital revolution is more and more entering routine diagnostics and assists pathologists in the evaluation of HE and immunohistological stainings 47-49, the applied morphome represents a conceivable algorithm for a multiparametric EMT based predictive model, capable of automation.

Nevertheless, two main limitations of our study concept have to be discussed. First, the investigated hotspot area was smaller than the internationally recommended field size. ITBCC guidelines recommend to assess tumor budding in one hotspot measuring 0.785 mm^2^ at the invasive front. 13 In our study, due to the resolution and size of the used monitor, only 0.517 mm^2^ visible area of the digital slide was available for manual quantification of tumor buds at the recommended 20× objective magnification. Nevertheless, Lohneis et al. demonstrated that, regardless of the applied quantification approach, an increased number of tumor buds correlates with a poor patient survival. The second limitation is the usage of ECad as an immunohistological marker for a) EMT and b) the facilitated detection of epithelial cells (together with histological cell morphology). As being a reliable marker for EMT, 29,30 recent literature lacks publications combining tumor bud quantification and ECad expression. Nevertheless, by the dual usage of ECad immunohistochemistry and histology no ECad negative tumor cell was detected.

Taken together, we established a multivariable model of tumor budding and ECad shifting in periampullary adenocarcinomas. We first investigated their relationship in the complex morphomic context, confirming prognostic relevance of this process. Our results also call attention to the fact that the prognostic value of the larger tumor cell clusters can be as high as that of the small tumor buds. We can conclude that tumor budding of periampullary cancers can be used in prognostic stratification of these tumors and may assist therapeutic decision making in the future.

## Supplementary Material

Supplementary figures and tables.

## Figures and Tables

**Table 1:** Cohort characteristics

Variable	n/%
Age at surgery (median, range)	67 (30-89)
**Sex**	
male	88 (51.5%)
female	83 (48.5%)
**Pathologic stage (pT)**	
pT1	13 (7.6%)
pT2	27 (15.8%)
pT3	113 (66.1%)
pT4	18 (10.5%)
**Regional lymph nodes (pN)**	
pN0	57 (33.3%)
pN+	114 (66.7%)
**Distant metastases (M)**	
M0	164 (95.9%)
M+	7 (4.1%)
**Lymphovascular invasion**	
Present	74 (43.3%)
Absent	97 (56.7%)
**Vascular invasion**	
Present	26 (15.2%)
Absent	145 (84.8%)
**Perineural invasion**	
Present	101 (59.1%)
Absent	70 (40.9%)
**Residual tumor**	
R0	126 (73.7%)
R+	45 (26.3%)
**Histologic grade**	
Grade 1	3 (1.8%)
Grade 2	108 (63.2%)
Grade 3	59 (34.5%)
Grade 4	1 (0.6%)
**Tumor localization**	
PDAC	110 (64%)
DBDAC	18 (10.5%
AMPAC	36 (20.9%)
DUOAC	7 (4.1%)
**Histologic subtype**	
PB	104 (60.8%)
MIX	11 (6.4%)
INT	34 (19.9%)
UNDIFF	17 (9.9%)
OTH WHO	5 (2.9%)
Follow-up period (median/range)	16 (1-116)
Death	88 (51.5%)
Total cell number (median/range)	336 (16-1108)
Total cell cluster number (median/range)	42 (3-283)
Overall median cell cluster size (median/range)	16 (2-36)
**Selected morphome features (median/range)**	
buds.10	7 (0-32)
buds.35	1 (0-5)
cyto.15	0.2 (0-1)
cyto.30	0.1 (0-0.6)
budding grade	
low	86 (50.3%)
high	85 (49.7%)

PDAC: pancreas ductal adenocarcinoma; DBDAC: distal bile duct adenocarcinoma; AMPAC: ampullary adenocarcinoma; DUOAC: duodenal adenocarcinoma; PB: pancreatobiliary histology; MIX: mixed histology; INT: intestinal histology; UNDIFF: undifferentiated histology; OTH WHO: WHO other histological type; buds.10: number of tumor cell clusters containing 6-10 cells; buds.35: number of tumor cell clusters containing 31-35 cells; cyto.15: fraction of cells with cytoplasmic E-Cadherin expression in tumor cell clusters containing 11-15 cells; cyto.30: fraction of cells with cytoplasmic E-Cadherin expression in tumor cell clusters containing 31-35 cells.

**Table 2:** Multivariable model from selected prognostic morphome features

Variable	Hazard Ratio (CI)	*p*
cyto.15	12.797 (3.578-45.771)	<0.001
cyto.30	5.881(1.424-24.281)	0.014
buds.10	1.054 (1.02-1.088)	0.001
buds.35	0.709 (0.561-0.898)	0.004

buds.10: number of tumor cell clusters containing 6-10 cells; buds.35: number of tumor cell clusters containing 31-35 cells; cyto.15: fraction of cells with cytoplasmic E-Cadherin expression in tumor cell clusters containing 11-15 cells; cyto.30: fraction of cells with cytoplasmic E-Cadherin expression in tumor cell clusters containing 31-35 cells.

**Table 3:** Multivariable model for overall survival in all PAMPAC

Variable	Hazard Ratio	*p*
Age	1.019 (0.997-1.042)	0.09
Sex (male)	1.58 (0.982-2.54)	0.059
Distant metastasis	3.856 (1.519-9.792)	0.005
Lymphovascular invasion	1.616 (1.013-2.578)	0.044
MIX	0.539 (0.23-1.265)	0.156
INT	0.154 (0.052-0.458)	0.001
UNDIFF	0.84 (0.42-1.68)	0.622
WHO OTH	0.357 (0.109-1.171)	0.089
DBDAC	0.918 (0.474-1.778)	0.799
AMPAC	0.42 (0.215-0.822)	0.011
DUOAC	1.779 (0.477-6.636)	0.391
High budding grade	2.606 (1.653-4.108)	<0.001

PAMPAC: periampullary adenocarcinoma; DBDAC: distal bile duct adenocarcinoma; AMPAC: ampullary adenocarcinoma; DUOAC: duodenal adenocarcinoma; MIX: mixed histology; INT: intestinal histology; UNDIFF: undifferentiated histology; OTH WHO: WHO other histological type.

**Table 4:** Multivariable model for overall survival in all PDAC

Variable	Hazard ratio	*p*
Age	1.02 (0.995-1.045)	0.116
Lymph node metastasis	2.105 (1.1-4.028)	0.025
Distant metastasis	3.187 (1.085-9.363)	0.035
Vascular invasion	1.673 (0.886-3.159)	0.112
High budding grade	3.455 (1.908-6.257)	<0.001

PDAC: Pancreatic Ductal Adenocarcinoma.

**Table 5:** Survival analysis data according to budding grade

Group	n	Number of events	Median OS	*p*
**Total PAMPAC cohort**				
Low budding grade	86	35	52	<0.001
High budding grade	85	53	17	<0.001
**PDAC**				
Low budding grade	51	21	27	<0.001
High budding grade	59	37	14	<0.001

PDAC: pancreas ductal adenocarcinoma, PAMPAC: periampullary adenocarcinoma.
