# Supplementary material for: Prognostic impact of tumor budding and EMT in periampullary adenocarcinoma: a quantitative approach
Source: J Cancer. 2020 Sep 17;11(22):6474–83. doi: 10.7150/jca.46093 (PMC7545681; doi:10.7150/jca.46093)
Supplement: Supplementary file 1 — Supplementary figures and tables. [file jcav11p6474s1.pdf]

Supplementary tables

Supplementary table 1 – selected prognostic morphome features from elastic net regression

Variable description	Mean coefficient in ten cross-validation runs	Number of selections among ten cross-validation runs
**Fraction of cells with cytoplasmic positivity in 11-15 cell size group (cyto.15)**	**1.60**	**10**
**Fraction of cells with cytoplasmic positivity in 26-30 cell size group (cyto.30)**	**0.84**	**10**
**Number of cell clusters containing 6-10 cells (buds.10)**	**0.02**	**10**
**Number of cell clusters containing 31-35 cells (buds.35)**	**-0.15**	**10**

Elastic net regression was performed using the glmnet package[22] with the cox proportional hazards model.[23] To account for eventual instability, feature selection was repeated ten times with tenfold internal cross-validation and an alpha value of one. Features that were consistently and repeatedly (>80%) selected by the elastic net (non-zero coefficient under the optimal lambda tuning value) were used for further analysis.

Supplementary table 2 – Univariable comparison of budding categories

Variable	Low budding	High budding	*p*
Condition	n/%	n/%	
Age at surgery (median, range)	67 (30-84)	67 (40-89)	0.983
Sex			
male	47 (55%)	41 (48%)	0.493
female	39 (45%)	44 (52%)	
Pathologic stage (pT)			
pT1	5 (6%)	8 (9%)	0.741
pT2	15 (17%)	12 (14%)	
pT3	56 (65%)	57 (67%)	
pT4	10 (12%)	8 (9%)	
Regional lymph nodes (pN)			
pN0	30 (35%)	27 (32%)	0.787
pN+	56 (65%)	58 (68%)	
Distant metastases (M)			
M0	82 (95%)	82 (96%)	1
M+	4 (5%)	3 (4%)	
Lymphovascular invasion			
Present	35 (41%)	39 (46%)	0.596
Absent	51 (59%)	46 (54%)	
Vascular invasion			
Present	11 (13%)	15 (18%)	0.502
Absent	75 (87%)	70 (82%)	
Perineural invasion			
Present	50 (59%)	50 (59%)	1
Absent	35 (41%)	35 (41%)	
Residual tumor			
R0	63 (73%)	63 (74%)	1
R+	23 (27%)	22 (26%)	
Histologic grade			
Grade 1	1 (1%)	2 (2%)	0. 158
Grade 2	61 (71%)	47 (55%)	

Grade 3	24 (28%)	35 (41%)	
Grade 4	0 (0%)	1 (1%)	
Tumor localization			
PDAC	51 (59%)	59 (69%)	0.437
DBDAC	10 (12%)	8 (9%)	
AMPAC	22 (26%)	14 (16%)	
DUOAC	3 (3%)	4 (5%)	
Histologic subtype			
PB	49 (57%)	55 (65%)	0.136
MIX	6 (7%)	5 (6%)	
INT	23 (27%)	11 (13%)	
UNDIFF	7 (8%)	10 (12%)	
OTH WHO	1 (1%)	4 (5%)	
Follow-up period (median/range)	20 (1-116)	12 (1-106)	
Death	88 (51.5%)		
Total cell number (median/range)	314.5 (16-1108)	362(76-975)	0.166
Total cell cluster number (median/range)	34.5 (3-283)	47 (6-243)	0.001
Overall median cell cluster size (median/range)	20.5 (2-36)	13 (4-29)	<0.001
Selected morphome features (median/range)			
buds.10	5 (0-22)	7 (0-32)	<0.001
buds.35	1 (0-5)	0 (0-3)	<0.001
cyto.15	0.165 (0-0.54)	0.318 (0.061-1)	<0.001
cyto.30	0.1 (0-0.296)	0.155 (0-0.574)	<0.001

Supplementary table 3 – Descriptive statistic of cell clusters

	1-5 cells	6-10 cells	11-15 cells	16-20 cells	21-25 cells	26-30 cells	31-35 cells	36-40 cells	41-45 cells	46-50 cells
**Cell cluster size**										
Median	21.0	7.0	3.0	2.0	1.0	1.0	1.0	0.0	0.0	0.0
Mean	30.8	8.164	4.111	2.515	1.491	1.251	0.8187	0.5848	0.4094	0.4444
Range	1-273	0-32	0-22	0-10	0-6	0-7	0-5	0-3	0-8	0-3
**Number of cells**										
Median	52.0	52.0	42.0	37.0	24.0	27.0	31.0	0.0	0.0	0.0
Mean	65.78	63.19	52.3	44.51	33.92	34.89	26.78	22.5	17.58	21.19
Range	1-384	0-237	0-276	0-176	0-137	0-207	0-163	0-117	0-348	0-145
**Fraction of MEM cells**										
Median	0.08411	0.13253	0.1975	0.1429	0.0896	0.0667	0.0313	0.0	0.0	0.0
Mean	0.13047	0.19116	0.2251	0.1899	0.1670	0.1688	0.1388	0.1137	0.0865	0.0718
Range	0-0.7143	0-0.8	0-0.8333	0-0.9091	0-0.6957	0-0.8077	0-0.7879	0-0.8649	0-0.7845	0-0.7579
**Fraction of CYTO cells**										
Median	0.5256	0.3608	0.2500	0.1954	0.1600	0.0909	0.0286	0.0	0.0	0.0
Mean	0.5510	0.3756	0.2597	0.2046	0.1690	0.1251	0.0915	0.0603	0.0557	0.0564
Range	0-1	0-0.9194	0-1	0-1	0-0.9535	0-0.5741	0-0.75	0-0.7692	0-0.7	0-0.9387
**Fraction of MIX cells**										
Median	0.3391	0.4286	0.5000	0.5200	0.4583	0.3966	0.2812	0.0	0.0	0.0
Mean	0.3186	0.4157	0.4918	0.4535	0.4009	0.3435	0.3020	0.2353	0.1853	0.1876
Range	0-1	0-1	1-0.9070	0-0.9375	0-1	0-1	0-0.9143	0-0.9211	0-0.8571	0-0.9792

Supplementary table 4. Inverse regression analysis of cluster count versus cluster size

parameter	term	coefficient	standard error	statistic	p
Cluster count	constant	-4.613	0.396	-11.658	<0.0001
	1/cluster size	165.107	5.026	32.849	<0.0001
Cytoplasmic	constant	0.128	0.008	16.825	<0.0001
	1/cluster size	2.187	0.081	26.903	<0.0001
Mixed	constant	0.6	0.009	69.907	<0.0001
	1/cluster size	-1.468	0.092	-15.983	<0.0001
Membranous	constant	0.272	0.009	30.795	<0.0001
	1/cluster size	-0.719	0.094	-7.615	<0.0001
